# Effects of Supplementing Finishing Goats with *Mitragyna speciosa* (Korth) Havil Leaves Powder on Growth Performance, Hematological Parameters, Carcass Composition, and Meat Quality

**DOI:** 10.3390/ani12131637

**Published:** 2022-06-26

**Authors:** Pin Chanjula, Juraithip Wungsintaweekul, Rawee Chiarawipa, Kampanat Phesatcha, Chanon Suntara, Rittikeard Prachumchai, Patcharin Pakdeechanuan, Anusorn Cherdthong

**Affiliations:** 1 Animal Production Innovation and Management Division, Faculty of Natural Resources, Hat Yai Campus, Prince of Songkla University, Songkhla 90110, Thailand; 2 Department of Pharmacognosy and Pharmaceutical Botany, Faculty of Pharmaceutical Sciences, Hat Yai Campus, Prince of Songkla University, Songkhla 90110, Thailand; juraithip.w@psu.ac.th; 3 Agricultural Innovation and Management Division, Faculty of Natural Resources, Hat Yai Campus, Prince of Songkla University, Songkhla 90110, Thailand; rawee.c@psu.ac.th; 4 Department of Animal Science, Faculty of Agriculture and Technology, Nakhon Phanom University, Nakhon Phanom 48000, Thailand; kampanatmon@gmail.com; 5 Tropical Feed Resource Research and Development Center (TROFREC), Department of Animal Science, Faculty of Agriculture, Khon Kaen University, Khon Kaen 40002, Thailand; chansun@kku.ac.th (C.S.); rittikeard1994@gmail.com (R.P.); anusornc@kku.ac.th (A.C.); 6 Division of Food Science and Nutrition, Faculty of Science and Technology, Pattani Campus, Prince of Songkla University, Pattani 94000, Thailand; patcharin.p@psu.ac.th

**Keywords:** *Mitragyna speciosa* (Korth) Havil leaves, growth performance, meat quality, goats

## Abstract

**Simple Summary:**

*Mitragyna speciosa* is an herbaceous plant found mostly in Thailand’s southern regions. This plant has phytonutrient elements that may be beneficial to ruminants. We observed that giving Mitragyna leaf powder to growing goats improved meat quality by increasing hot carcass weight, longissimus muscle area, oleic acid content, and protein content. As a result, Mitragyna leaf supplementation for small ruminant production may become a viable alternative feed additive in the future.

**Abstract:**

The objective of this study was to see how dried *Mitragyna speciosa* Korth leaves (DKTL) affected growth, hematological parameters, carcass characteristics, muscle chemical composition, and fatty acid profile in finishing goats. In a randomized complete block design, twenty crossbred males (Thai Native x Boer) weaned goats (17.70 ± 2.50 kg of initial body weight (BW)) were provided to the experimental animals (5 goats per treatment) for 90 days. Individual dietary treatments of 0, 2.22, 4.44, and 6.66 g/d of DKTL on a dry matter basis were given to the goats. The diets were provided twice daily as total mixed rations ad libitum. In comparison to the control diet, DKTL supplementation had no effect on BW, average daily gain (ADG), feed conversion ratio (FCR), carcass composition, meat pH, or meat color (*p* > 0.05). After DKTL treatment, the hot carcass weight, longissimus muscle area, oleic acid (C18:1n9), monounsaturated fatty acid (MUFA), and protein content increased, but saturated fatty acids (SFA) and ether extract decreased (*p* < 0.05). To summarize, DKTL supplementation can improve goat meat quality.

## 1. Introduction

The world’s rapidly growing population, along with the widespread opinion that animal products are advantageous to human health, has increased the demand for animal products dramatically. However, because red meat has a low proportion of beneficial polyunsaturated fatty acids (PUFA) and is high in saturated fatty acids (SFA), some consumers believe it is undesirable [1]. Researchers have investigated the use of herbs or phytogenic feed additives to increase animal production throughout the last decade [2]. Incorporating phytogenic feed additives into a total mixed ration in goats has been shown to improve PUFA levels considerably [3]. Furthermore, these supplements have been proven in agricultural livestock to promote feed intake, nutrient utilization, immune system modulation, and endocrine system stimulation, resulting in better nutrient utilization, growth performance, and animal health [1].

Phytogenic feed additives or phytonutrients such as phenolic compounds (PCs), condensed tannins (CT), saponins (SP), and essential oils (EOs) are examples of plant secondary metabolites that play an important role in animal nutrition and health [2,4] and have been studied as a natural rumen modifier [5,6]. Furthermore, those substances have an influence on antioxidant capacity, which may defend the body against the availability of free radicals [7]. Free radicals and reactive oxygen species are continually produced by animals’ bodies [8]. A range of endogenous antioxidant enzymes defends mammals against reactive oxygen species or free radicals in general. However, animals from tropical areas are more sensitive to oxidative stress as a result of their constant exposure to high temperatures [9]. In order to better understand the role of oxidant and antioxidant molecules in physiological settings in ruminant nutrition, there has been an increase in interest in studies on oxidative stress, which is defined as an imbalance between oxidants and antioxidants [10]. Natural antioxidants derived from a variety of plant sources have been promoted as a defense against lipid and protein oxidation in meat products as well as the implications for meat quality, which have been demonstrated to be comparable to or better than synthetic anti-oxidants [11].

Methane (CH_4_) is one of the secondary metabolites created during ruminal digestion since the animal is unable to profit from hydrogen (H_2_) and carbon dioxide (CO_2_) production during fermentation. Methanogen bacteria use hydrogen and CO_2_ to generate CH_4_ [12,13]. Additionally, up to 12% of the feed energy may be lost as emitted CH_4_ [14,15]. Furthermore, because some plants contain secondary metabolites, such as saponins, which are the principal components of Yucca extract [16], plant extracts and essential oils have a special potential in this field [17,18]. They can affect animal production, ruminal fermentation, and digestion, despite the fact that they are not considered nutrients in and of themselves.

*Mitragyna speciosa* (Korth.) Havil. is a tropical tree native to Malaysia, Indonesia, and Thailand. It is an indigenous tropical tree in Thailand known as Kratom, and it belongs to the Rubiaceae plant family. Its leaves have been used in folk medicine (at low doses) for hundreds of years to combat tiredness and promote tolerance to hard work in the blazing heat [19,20]. Kratom leaves are a non-toxic and physiologically safe alternative source of plant bioactive or PSC components such as alkaloids, polyphenols, tannins, flavonoids, saponins, and antioxidants. *M. speciosa* contains a total phenolic content of 4.0–4.1%, a flavonoid content of 11.2–19.4%, and a mitragynine concentration of 4.1–7.0%, according to Goh et al. [21] and Chanjula et al. [22]. The central nervous system stimulant and depressive properties of Kratom leaves have been identified. PSCs have also been shown to affect ruminal fermentation, animal performance, animal health, and animal product quality [23,24]. Antioxidants in bovine feed have been shown to minimize oxidative stress and increase plasma antioxidant potential [25]. Additionally, those herbs contain phytonutrients such as CT and SP, which improve milk production and quality, nitrogen utilization in dairy cows [26], and rumen fermentation [27], as well as a protective effect on ruminal protein to increase duodenal consumption and change the volatile fatty acid ratio in the rumen [28].

DKTL has been studied as a supplement for goats, and it has been discovered that these products can lower ammonia nitrogen (NH_3_-N), blood urea nitrogen (BUN), cholesterol (CHOL), low-density lipoprotein (LDL), and triglyceride (TG) levels while increasing high-density lipoprotein (HDL) levels. Probably owing to high levels of mitragynine or secondary metabolites, which are assumed to be induced by direct inhibition of cholesterol, LDL, and triglyceride synthesis in the liver, whilst protozoa population and CH_4_ production fell linearly, but propionic acid (C3) production rose linearly [22]. On the other hand, the effects of DKTL on matured goats’ growth performance, hematological parameters, and carcass features have yet to be investigated. The bioactive components in DKTL were thought to increase feed efficiency and rumen ecology, leading to higher goat productivity and better carcass quality. As a result, the goal of this study was to see how supplementing finishing goats with *Mitragyna speciosa* leaf powder affected their growth performance, hematological parameters, and meat quality (Thai Native × Bore).

## 2. Materials and Methods

### 2.1. Preparing of Dried Kratom Leaves (DKTL) for Animals

In October 2021, fresh Kratom leaves were harvested in Tambon Namphu, Ban Na San District, Surat Thani Province, Thailand. The plant sample was authenticated at Prince of Songkla University’s Department of Biology, Faculty of Science, Songkhla, Thailand, where the herbarium vouchers (N5/001 (PSU)) were kept. Ripe Kratom leaves were rinsed in distilled water after being washed in running tap water. It was then dried for 72 h at 45–50 °C and processed in a Cyclotech Mill (Tecator, Haganäs, Sweden) to generate Kratom leaf powder, which was then packaged in airtight polyethylene bags and stored in a cold, dry environment as advised by Hagerman and Butler [29]. 

### 2.2. Preparation of the Extract and Chemical Composition

Using the procedures described by Jamil et al. [30], the alkaloids were extracted and isolated from the plant. DKTL samples were tested for CT and SP using a modified Vanillin-HCL technique [31]. The Association of Official Agricultural Chemists [32] used dry matter (DM, ID 967.03), organic matter (OM, ID 942.05), crude protein (CP) (ID 984.13), ether extract (EE, ID 920.39), acid detergent fiber (ADF), and neutral detergent fiber (NDF) methods to determine the chemical composition of DKTL [33].

### 2.3. Animals, Experimental Design, Diets, and Feeding

The research was conducted at the Faculty of Natural Resources, Prince of Songkla University’s Experimental Goat Farm in Hat Yai City, Songkhla Province, Thailand, 90112. Because the Animal Farm Department has a limited number of animals with the same condition, it is difficult to provide more animals for the current inquiry (breed, BW, age, etc.). As a result, only a small number of animals were employed in the experiment. This work, however, was done with prudence and accuracy to avoid any mistakes caused by humans, animals, or the environment. Twenty uncastrated developing male goats (17.70 ± 2.5 kg of beginning BW and 4 months with 15 days of age) were split and blocked by BW to receive four different dosages of DKTL in a randomized full block design. Within each block, the goats were randomly allocated to one of four treatments (5 goats per treatment) and allowed 14 days to adapt to the experimental facilities and feed before data collection. During the adjustment period, all animals were vaccinated, parasite-treated, and given vitamin A, D, and E injections. Individually ventilated cages (1.2–0.8 m) with wooden slotted flooring were housed in an open goat barn erected above ground, with constant access to water and mineral salt. The animals were weighed after a 16-h fast to determine their original body weight after adaptation (IBW). All animals received their own total mixed ration (TMR, Table 1) meals throughout the trial, which were planned to be isonitrogenous and isocaloric (DM basis) in order to fulfill or exceed the NRC [34] requirements for fattening goats with an average weight gain of 200 g/day. The composition of DKTL is shown in Table 2 and daily DKTL supplementation at 0, 2.22, 4.44, and 6.66 g/goat/day (on a DM basis) for 90 days were assigned at random to the following treatment sequences. 

Total mixed rations (TMR) included 30% Pangola grass hay (PGH), 36.2 percent ground maize, 22.7 percent soybean meal, 0.5 percent fish meal, 4.0 percent Leucaena a leaf meal, 5% molasses, 0.3 percent dicalcium phosphate, and 1% mineral mix.

To ensure that all supplements were ingested by the animals, DKTL was hand-mixed with 200 g of TMR feed before the morning feeding, and extra TMR was provided to individual animals later. Feed was given ad libitum in two equal doses at 7:00 a.m. for 90 days. To guarantee ad libitum feed consumption, the feed intake rate was changed to generate orts of around 10% of the intake between 2:00 and 4:00 p.m. To precisely compute dry matter intake (DMI), feed orts were weighed daily, verified for DM, documented, and destroyed. Weekly DM evaluations of individual items were conducted in order to adjust the diet composition for component moisture content. Weekly composite feed samples were collected for DM analysis and dried for 48 h in a forced-air oven at 60 °C. Before being analyzed for DM, CP, ether extract, and ash content, samples were crushed to pass through a 1-mm screen (Cyclotech Mill, Tecator, Hoganas, Sweden) [32]. Van Soest et al. [33] designed methods to evaluate neutral and acid detergent fibers, and calculated non-fibrous carbohydrate (percent in DM) as 100 − (CP + NDF + ether extract + ash) [35].

### 2.4. Animal Performance, Slaughter, and Sample Collection

All animals were weighed using a digital scale before being transferred to the Institute of Animal Science’s slaughterhouse at the start of the study and every 2 weeks thereafter at the same time (final weight). The DMI of each goat was computed by multiplying their weekly intake by the number of days in the week. The average daily gain (ADG) was calculated by dividing the BW gain by the number of days in the trial. The feed conversion was calculated using the ADG to DMI ratio (g of BW gain/g of DMI).

Blood samples (about 10 mL each) were taken from the jugular vein at 0 and 4 h post-feeding on day 89 in tubes containing 12 mg of EDTA on the last day of the data collection period. In the plasma, hematocrit, erythrocytes, leukocytes, hemoglobin, and differential leucocytic count were all measured before it was analyzed (lymphocytes, basophils, eosinophils, monocytes, and neutrophils). During the experiment, the Wintrobe formula was utilized to compute hematological parameters such as mean corpuscular volume (MCV) and mean corpuscular hemoglobin (MCHC). Another blood sample was taken and put in a tube (without EDTA) at room temperature for 20 min before being centrifuged at 3000 rpm for 10 min to separate the serum (Table Top Centrifuge PLC-02, Enfield, CT, USA). The serum was kept at −20 °C until mineral estimation and biochemical analyses were performed (within one day). Total protein, albumin, creatine kinase, urea, and glucose levels were measured in a spectrophotometer using commercial kits. Hemoglobin and non-esterified fatty acids (NEFA) were measured using Bioclin commercial kits (Bioclin^®^, Belo Horizonte, Brazil). 

When the animals reached 90 days, they were murdered in the slaughterhouse after an overnight period without food but with unrestricted access to water, as to Thai Agricultural Conventional TAS 6006 and conventional slaughter protocols [36]. Before and after slaughter, the weights of fasted live and hot carcasses were measured. Non-carcass components (skin, head, feet, lung, heart, liver, spleen, kidneys, kidney fat, and gastro-intestinal tract fat) were removed and weighed after the animals were slaughtered. The stomach (rumen, reticulum, and omasum) was removed and weighed separately, as was the post ruminal tract (small intestine, large intestine, and caecum). The contents of the stomach and post ruminal tract were removed, cleaned, and weighed to estimate the weight of the empty stomach and post ruminal tract.

The proportion of carcass production was calculated. The carcasses were chilled at 4 °C for 24 h, and the cold carcass weight was estimated the next morning. The carcass was measured in length and breadth. The bodies were sliced in half lengthwise. The right sides of the carcasses were split into eight portions (loin, hind leg, chump, rack, should, foreleg, breast, and neck) and weighed individually, according to Thai Agricultural Standard TAS 6006 [37]. After that, each portion was separated into lean meat, bone, and trimmings, and weighed separately. A muscular longissimus thoracis (LT) area was generated between ribs 12 and 13 on the left sliced surface (of the chilled carcass). The LT was taken (the region of the spine between the last lumbar and first sacral vertebrae). The fatty acid profile, chemical composition, flesh color, and shear force parameters of these two parts of meat per animal were immediately labeled and frozen for subsequent testing.

### 2.5. Meat Chemical Analyses

AOAC [32] standard techniques were used to perform proximate analysis on feed and LT muscle samples. Dry matter (DM) was determined by drying in a forced-air oven for 24 h at 105 degrees Celsius. The N content of feed and LT muscle was determined using a Kjeltec Auto Analyzer (Tecator, Hoganas, Sweden), and the ether extract (EE) in petroleum ether was determined using a Soxtec Auto Analyzer (Tecator). The ash composition of the samples was evaluated by ashing them in a muffle furnace for 5 h at 550 °C.

### 2.6. Meat pH, Temperature, Color, and Physical Properties

The initial pH and temperature were measured between the 12th and 13th ribs in the center of the LM 45 min after slaughter using a digital penetration pH meter model AG 8603 (Mettler Toledo, AG 8603, Schwerzenbach, Switzerland) after calibration with two buffers (7.00 and 4.01) and Thermohydrometers (HI9564, Hanna Instrument (Thailand) Co., Ltd. Bangkok, Thailand). After the corpse had been chilled, the pH and temperature were tested at the same site (24 h postmortem). Meat color, conductivity, water loss during cooking, water retention capacity, and shear force were all assessed on this piece of the loin.

A Hunter Lab Miniscan Plus Spectrocolorimeter was used to objectively assess the color of the muscle surface on the same sliced surface as the LT. To acquire a typical reading for L* (darkness to lightness; lower L* implies a dark color), a* (redness; higher a* value indicates a redder color), and b* (yellowness; higher b* value indicates a yellower hue), instrumental color measurements were performed at three regions of exposed lean.

Defrosting samples at room temperature until their inner temperatures reached 2 to 5 °C was used to assess shear force. After weighing the samples, thin sections from the lateral and extremities were removed; four samples, each 1 cm thick and 5 cm long were obtained to measure shear force in a texture analyzer (TA-XTPlus-Texture Analyzer, with a Warner-Bratzler Blade probe, Texture Expert Exponent-Stable MicroSystems software, Ltd. in Godalming, Surrey, UK) [38]. Each sample had ten shear force measurements taken. The AOAC [32] standard techniques were used to examine LT muscle samples proximally.

To calculate water loss, the meat was weighed and grilled on an electric grill. The loin pieces were wrapped in aluminum foil and used to make the meal. The steaks were flipped over and grilled until the second side reached 71 °C, or when they reached 40 °C at their coldest point. Before being weighed again, the meat pieces were allowed to cool to room temperature [39]. The raw longissimus muscle that remained after the steaks were removed for frying was tested for its ability to retain water. These samples were centrifuged at 1500 rpm for 4 min before being baked at 70 °C for 18 h. The water holding capacity was calculated using the weight differential [39].

### 2.7. Fatty Acids Profile Analysis

The procedures for extracting and methylating fatty acids from meat developed by Hara and Radin [40] were applied. After extraction and methylation, each sample was injected (1 mL) onto a Finnigan GC Focus gas chromatograph (Model HP 6890, Hewlett-Packard Co., Ltd., Rochester, NY, USA) with a flame ionization detector, capillary column CP-Sil 88 (Varian), and 100-m length, 0.25-m diameter, and 0.20-m thick film. The carrier gas was hydrogen, which flowed at a rate of 1.8 mL/min. For a total of 65 min, the oven temperature was kept at 70 °C for 4 min, then increased to 175 °C (13 °C/min) for 27 min, 215 °C (40 °C/min) for 9 min, and lastly rose to 230 °C and held for 5 min. The vaporizer was set to 250 °C, while the detector was set at 300 °C. The retention duration of the samples’ methyl esters was matched to a preset pattern, and the fatty acids were measured by normalizing the methyl esters’ areas; the findings are presented as percentage areas.

### 2.8. Statistical Analysis

SAS (Cary, NC, USA) [41] software was used to analyze all of the data. The MIXED technique was used to investigate the fixed effects of treatment and block on performance and carcass characteristics using animals as the experimental unit. The linear, quadratic, and cubic effects, as well as the effect of 0 g/d DKTL vs. the average of all diets comprising 2.22–6.66 g/d DKTL, were determined using orthogonal contrasts. Treatment effects on response variables were investigated using orthogonal polynomial contrasts (linear and quadratic). *p* < 0.05 and trends (0.05 > *p* ≤ 0.10) were used to determine significance. The standard errors of the mean are calculated and given.

## 3. Results and Discussion

### 3.1. Chemical Composition of Feeds

For DM, CP, ash, OM, EE, NDF, ADF, and ADL, the DKTL had nutritive values of 95.24%, 20.10%, 4.11%, 95.89%, 1.71%, 44.49%, 27.31%, and 8.25%. Phesatcha et al. [42] observed that *Mitragyna speciosa* leaf powder (MSLP) contained 94.3% OM, 21.2% CP, 51.4% NDF, and 28.2% ADF, respectively. In DKTL, the concentrations of mitragynine, CT, and SP were 4.14%, 8.28%, and 5.21%, respectively. Similarly, Goh et al. [21] and Phesatcha et al. [42] discovered mitragynine concentrations of 6.53–8.20%, CT concentrations of 14.6%, and SP concentrations of 12.1% in *Mitragyna speciosa*. This study’s mitragynine concentration was identical to that observed by Kikura-Hanajiri et al. [43], who discovered that the optimal range for mitragynine was 1.2 to 6.3% and for 7-HMG was 0.01 to 0.04%. DKTL has a lot of CT, especially when compared to other tropical fruits and plants, including dragon fruit peel powder (which has 6.9% CT), *Flemingia macrophylla* (which has 5.6% CT), and *Leucaena lucocephala* (which has 3.6% CT) [44,45,46]. While the concentrations of other phytonutrients or PSCs were lower, MSLP had a total phenolic of 407.83 GAE mg/g and flavonoids of 194.00 QE mg/g, which was much lower than what Goh et al. [21] observed. 

### 3.2. Feed Intake, Performance, Carcass, and Meat Traits

The effect of DKTL on the growth performance of finishing goats is shown in Table 3. DKTL supplementation had no negative impacts on final body weight, weight increase, dry matter intake, nutrient intake (OM, CP, NDF, and ADF), average daily gain (ADG), or feed conversion ratio (FCR). This study’s 16.46% crude protein and 2.75 Mcal/kg DM metabolizable energy were likely capable of maintaining live weight and growth across all treatments. In this study, an ADG of 0.138 to 0.157 kg/d was detected, which is higher than the ADG of 0.0496 kg and 0.0818 kg/d discovered by Johnson et al. [47] in animals fed forages and cereals, respectively. This might be due to the fact that DKTL includes bioactive compounds with a variety of modes of action on digestive processes. The majority of bioactive compounds discovered in herbs increase bile acid synthesis and excretion in the liver [48]. In the small intestine, bile is required for fat breakdown and absorption. DKTL polyphenols work as enzyme inducers, binding to enzymes and activating them for a variety of metabolic functions in the body, including digestion and nutrition absorption [49].

Our findings are similar to those of Chanjula et al. [22], who fed male crossbred (Thai Native × Anglo Nubian) goat’s diets with varying amounts of DKTL supplementation (up to 4.44 g/d) in recent research. According to the findings, mitragynine, CT, SP, and other PSCs in DKTL had a beneficial influence on nutritional digestibility. Similar findings were found by Vicknasingam et al. [50], who found that Kratom users experienced increased appetite (57.8–77.8%). Sultana et al. [51] and Su and Chen [52] found that high levels of CT and SP in *Moringa oleifera* impair nutritional digestibility, but only when 100 mg of *Sanguisorba officinalis* is added [53]. Mirzaei-Aghsaghali et al. [54] reported that lambs fed a diet enriched with *Planatago lanceolata* L. and garlic leaf gained more weight. Furthermore, increased growth rates in all herbal-supplemented groups might be connected to reduced parasite counts, decreased nutrient loss, and higher nutrient turnover. Supplementing with different herbs increased nutrient utilization by stimulating cellulolytic bacterial activity in the rumen while decreasing protozoa and methanogenic bacterial proliferation, resulting in less energy loss attributable to CH_4_ emission [55,56,57,58,59] The lowest response was observed at 6.66 g/d DKTL supplementation. However, the reasons for the low feed intake remain unexplained in the current inquiry into the slightly reduced nutritional digestibility observed when DKTL concentrations were raised over the threshold of 4.44 g/d. DKTL is thought to include anti-nutritional compounds such as mitragynine, CT, SP, and phenolic acids, which might affect animal digestion, metabolism, and nutrient absorption [22], and this could be linked to the DKTL diet’s lower palatability compared to the control diets. Additional study is needed to determine the cause of the lower feed intake before DKTL powder may be recommended as a feed supplement.

Dietary treatments showed no effect on the pH of LM muscle at 45 min or 24 h, which was within the range [60,61] (Table 4). The pH of the muscle after slaughter is one of the most important factors affecting meat quality since it impacts tenderness, color, and water retention capacity. Lactic acid production and accumulation accompany the depletion of muscle glycogen reserves [62]. Furthermore, rigor mortis occurs in sheep and goat carcasses when the pH of the longissimus muscle is between 5.6 and 5.8. The pH of chilled meat at the end of its shelf life, color, and quality are important. High pH has been associated with ruminant malnutrition and general stress, and such meat is often black in color [63]. 

Color is an important meat quality factor since customers’ first impression of any beef product is based on its color [64,65,66,67]. L*, a*, and b* were found to have average values of 39.14 to 39.44, 16.16–18.42, and 5.26 to 5.63, respectively, in this study. Goat meat exhibited L* values ranging from 30.6 to 39.9, a* values ranging from 11.8 to 18.0, and b* values ranging from 3.3 to 11.54, according to Chanjula et al. [68]. The absence of differences in carcass features can be explained by the constancy of the animals at the start of the feedlot period, as well as the same laughter weight. Compared to lamb meat, goat meat is darker, yellower, and has a higher a* value [69]. According to Priolo et al. [63], lambs fed a diet containing carob pulp had higher L* values than lambs fed a diet supplemented with polyethylene glycol (PEG; control). In previous experiments on small ruminants, CT-containing diets have been shown to alter the color of lamb meat [70]. When the LM of meat goats in our study was compared to the values for Boer-crosses reported by Solaiman et al. [71], the hunter colorimetric co-ordinate L* value was higher, and both a* (11.8 vs. 16.8) and b* (11.2 vs. 16.1) values were lower. This might be due to breed and food discrepancies between the current research and others [71].

The effects of DKTL on carcass width and longissimus muscle area were substantial (*p* < 0.01). When goats were given DKTL, they had higher values (*p* < 0.02) than the control group. When DKTL was introduced to the diet, it decreased drip loss and cooking loss. The Warner Bratzler shear force (WBSF) increased when DKTL was introduced to the diet. Despite this, the WBSF findings (4.0 kg) give softness, which may contribute to favorable consumer acceptance [72]. For both trained panelists and consumers, WBSF levels of more than 5.5 kg are typically seen as objectionably difficult [73].

The effects of treatment diets on the carcass of goats are shown in Table 5. In the DKTL supplementation groups, mortality was lower (quadratic; *p* < 0.05) than in the control group. It is unclear if the dietary therapy contributed to this. The current results cannot be compared to other results since there is insufficient data on the impact of DKTL supplementation on carcass cut. The current study’s findings, on the other hand, demonstrate that giving DKTL supplements to goats had no deleterious effect on their commercial cut. DKTL supplementation had no influence on the chemical composition of DM, ash, calcium, or phosphorous in LT among treatments since it was designed that way (Table 6). Dietary supplementation with DKTL had a beneficial effect on EE (L, *p* = 0.03). It is worth noting that CP was quantitatively higher (*p* < 0.01) in the animals given DKTL supplementation than in the control group. As stated by Beserra et al. [74], the chemical composition of the longissimus muscle ranges from 22.3 to 24.0, 20.5 to 21.9, 1.5 to 2.7, and 1.0 to 1.1, with DM, ash, CP, and EE levels ranging from 22.3 to 24.0, 20.5 to 21.9, 1.5 to 2.7, and 1.0 to 1.1, respectively. Previously, glucose was reported to be the primary lipid precursor in intramuscular adipose tissue, whereas acetate provided the highest proportionate contribution to lipogenesis in subcutaneous adipose tissue [75].

### 3.3. Fatty Acid (FA) Profiles in Longissimus dorsi Muscle of Fattening Goats

The effects of the treatment diets on the effluent FA concentration in LT are shown in Table 7. With the exception of C12:0, C14:0, C16:1, C18:3n3, and C20:4n6, DKTL supplementation had no influence on effluent FA composition. Nonetheless, there is no information on how animals scored in the DKTL feeding research.

Fatty acids are the main component of lipids, and they have an impact on the quality of meat. The saturation level of fatty acids is determined by their concentration, which has a substantial influence on their quality. Diet has an influence on the fatty acid makeup of biological tissues, as indicated by goats fed pasture having more unsaturated intermuscular fat than those fed grain [76]. Long-chain SFAs have a detrimental influence on meat palatability when they solidify quickly.

When compared to the control diet (no DKTL), the 16:0 and 18:0 ratios in the diets decreased linearly (*p* < 0.01) as DKTL was added. C18:1n9 concentrations in goats given DKTL rose linearly (Q, *p* < 0.01), with a trend to increase C16:1 (L, *p* = 0.09). It is likely that adding DKTL to your diet will increase the quality of your meat. Regardless, it is unclear if the nutritional therapy was to blame. The fatty acid composition of fat depots is assumed to closely follow that of meals [77]. Despite this, the current study identified only two changes in saturated and one unsaturated FA. These variations in FA composition appear to have an impact on the nutritional content of goat meat-fed glycerin up to 200 g/kg of concentrate. In the current study, there was a trend toward reduced muscle fat content when DKTL supplementation was higher than 2.22 g/d (Table 7).

The decreases in palmitic acid (C16:0) and stearic acid (C18:0), as well as the increase in oleic acid (C18:1n9), are notable because C16:0 is thought to be harmful to serum cholesterol levels, whereas C18:0 has been shown to have a net neutral effect on serum cholesterol in humans (Yu et al., 1995). C16:0 raised blood cholesterol levels, C18:0 had no impact, and oleic acid (C18:1n9) lowered blood cholesterol levels, according to Banskalieva et al. [78]. When goats provided with DKTL supplementation in meals were compared to goats who did not receive DKTL supplementation in their diets, oleic acid (C18:1n-9 cis) had a positive impact (L, *p* < 0.01) and was quantitatively higher. Oleic acid has been discovered as the most abundant fatty acid in lamb [79]. Greater amounts of this fatty acid in humans have been associated with higher levels of HDL (high-density lipoprotein), a beneficial cholesterol [80]. Banskalieva et al. [78] have suggested that the (C18 + C18:1)/C16 ratio might be useful in understanding the possible health effects of different lipid types. In the muscles, DKTL treatment enhanced monounsaturated fatty acid (C18:1) levels (*p* < 0.01).

### 3.4. Blood Metabolite

The blood biochemical parameters of goats given various dosages of DKTL-supplemented diet are shown in Table 8. Albumin, globulin, A:G ratio, Serum glutamic oxaloacetic transaminase (SGOT), Serum glutamate-pyruvate transaminase (SGPT), and alkaline phosphatase (ALP) were measured at 3.57–3.75 (g%), 2.39–2.65 (g%), 1.42–1.53 (g%), 88.67–109.00 (U/L), 21.17–26.33 (U/L), and 243.17–420.33 (U/L), respectively, with no effect of adding DKTL to the diet (*p* > 0.05). The total protein content of the food varied between 6.01–6.22 (g/dL), indicating that it is sufficient to maintain the animals’ development without jeopardizing their health. Despite this, all of the serum parameter values were similar to those previously reported by Chanjula et al. [22] and Ladokun et al. [81]. Furthermore, the SGOT, SGPT, and ALP readings dropped as the amount of DKTL added to goat rations increased, indicating that the goat’s liver integrity was conserved. Because the anti-nutrients in DKTL had no impact on the animals, the test material was considered acceptable for use in goat diets. 

RBC, Hb, WBC, MCV, MCH, MCHC RDW-CW%, NEU, LYMPH, and MONO levels did not differ significantly across treatments (*p* >0.05). Quintavalla et al. [82] indicate that hematology is an important tool for disease and nutrition verification. RBC, Hb, WBC, MCV, MCH, and MCHC levels varied from 3.25–3.57 (10^6^/ µL), 10.38–11.63 (g/dL), 13.37–17.65 (10^3^/ µL), 79.72–94.13 (fL), 29.27–34.25 (pg), and 35.85–37.53 (g/dL), respectively while the percentage of NEU, LYMP, and MONO were ranged as 38.67 (%), 48.67–50.67 (%), and 2.17–2.83 (%), respectively. However, data on the red blood profile and white blood profile of goats (such as RCB, Hb, MCV, Plt, RDW, and MCHC), WBC (NEU, LYM, and MONO), and TP (ALB and GLB) after DKTL supplementation are scarce. All of the obtained hematological values fell within Chanjula et al. [22]’s reference range for goats. According to Soetan et al. [83], hemoglobin aids in the transport of oxygen to animal tissues. The WBC and its differentials aid the body in fighting infections and producing antibodies to defend itself [84].

When compared to the control diet, supplementing with DKTL had no deleterious effects on lipid profiles and blood metabolites (Table 8 and Table 9), or plasma parameters (Table 10) (*p* > 0.05). Hematology indicators such as SGOT, Alp, Hb, and Plt were decreased with DKLT supplementation, but WBC was raised (*p* < 0.05).

According to Soetan et al. [83], hemoglobin aids in the transport of oxygen to animal tissues. Adeyinka and Bello [84] found that WBC and its differentials help in the fight against illnesses and the creation of antibodies to protect the body. A preliminary diagnosis of animal health concerns and abnormalities was made using blood metabolites. In cattle, serum GGT level has been shown to be a sensitive indicator of moderate hepatic impairment, whereas alkaline phosphatase and cholesterol are utilized to detect bile blockage and liver damage, respectively [85]. Nonetheless, in the current study, all of these variables had lower values than previously published data [86]. GGT (45 to 52 vs. 28.8 U/L), albumin (3.0 to 3.1 vs. 1.9 to 2.0 g/dL), and alanine aminotransferase (ALT) concentrations were lower (*p* ≤ 0.05) in the DKTL supplemented group when sheep were fed a basal supplemented diet with concentrate feedlot diets, according to Whitney et al. [87]. DKTL may be employed in goat supplementary diets without affecting feed consumption or animal health.

## 4. Conclusions

In this research, DKTL supplementation showed no detrimental impacts on BW, ADG, feed conversion ratio (FCR), carcass composition, meat pH, or meat color in this research as compared to the control diet. After DKTL treatment, the hot carcass weight, longissimus muscle area, fatty acid C18:1 n9, MUFA, and protein content all increased, while SFA and ether extract decreased. As a consequence, Mitragyna leaf supplementation as a feed additive for small ruminant production may become a feasible option in the future.

## Figures and Tables

**Table 1:** Dietary ingredients and chemical composition of total mixed ration (TMR) and Pangola grass hay (PGH) fed to the goats during the trial.

Item (% of DM)	TMR ^1^	
	Concentrate Diet	Roughage Source
Pangola grass hay (PGH)	-	30.0
Ground corn	36.2	-
Soybean meal	22.7	-
Fish meal	0.5	-
Leucaena leaf meal	4.0	-
Molasses	5.0	-
Dicalcium phosphate	0.3	-
Salt	0.3	-
Mineral and vitamin mix ^2^	1.0	-
Chemical composition, %		
Dry matter	91.69	94.26
% of DM		
Crude protein	16.46	3.18
Ash	5.92	5.65
Organic matter	94.08	94.35
Ether extract	3	1.99
Non-fibrous carbohydrate ^3^	31.92	14.77
Neutral detergent fiber	42.7	74.41
Acid detergent fiber	19.6	41.6
Acid detergent lignin	5.4	6.07
Gross energy, Mcal/kg DM	4.09	3.91
TDN, % ^4^	76.06	55.6
Metabolizable energy, Mcal/kg DM ^5^	2.75	2.01

^1^ TMR diet was divided into four treatments depending on DKTL supplementation level: T1 = 0.00, T2 = 2.22, T3 = 4.44, T4 = 6.66 g/goat per day of dried kratom leaves (DKTL). ^2^ Minerals and vitamins (each kg contains): Vitamin A: 10,000,000 IU; Vitamin E: 70,000 IU; Vitamin D: 1,600,000 IU; Fe: 50 g; Zn: 40 g; Mn: 40 g; Co: 0.1 g; Cu: 10 g; Se: 0.1 g; I: 0.5 g. ^3^ Calculated as: NFC = 100 − (% NDF + % CP + % EE + % ash). ^4^ Estimated by the equation TDN = (%) DCP + DNFC) + DEE 2.25 + (DNDF). ^5^ Estimated by the equation ME (Mcal/kg DM) = TDN 0.04409 0.82).

**Table 2:** Chemical composition and nutritive values of dried kratom leaves (DKTL) used in the experimental diets (on a dry matter basis) for goats.

Parameters	DKTL ^1^
Dry matter ^2^ (%)	25.45
Chemical composition (% of DM)	
Dry matter	95.24
Crude protein	20.1
Ash	4.11
Organic matter	95.89
Ether extract	1.71
Neutral detergent fiber	44.49
Acid detergent fiber	27.31
Acid detergent lignin	8.25
Gross energy, Mcal/kg DM	4.63
Alkaloid profile (%)	
Mitragynine	4.14
Paynantheine	0.59
Speciogynine	0.26
Total condensed tannin content (%)	8.28
Total saponin content (%)	5.21
Flavonoids (%)	11.24
Phenolic acids (%)	4.1
Antioxidant activity	
DPPH ^4^ (IC50 (mg/mL)	1.04
FRAP ^5^ (%)	3.98
Mineral profile ^3^	
Ca, %	0.84
P, %	0.2
K, %	1.53
Mg, %	0.3
S, %	1.26
Na, %	0.01
Fe, ppm	80.67
Cu, ppm	11.54
Mn, ppm	1862.3
Zn, ppm	32.14
B, ppm	69.71
Cr, ppm	3.23
Se, ppm	ND

^1^ Tambon Namphu, Ban Na San, Surat Thani Province, Thailand. ^2^ Fresh matter basis. ^3^ P: phosphorus; K: potassium; Ca: calcium; Mg: magnesium; S: sulfur; Na: sodium; Fe: iron; Cu: copper; Mn: manganese; Zn: zinc; Cr: chromium; Se: selenium. ND: not determined. ^4^ DPPH: 2,2-diphenyl-1-picrylhydrazyl. ^5^ FRAP: ferric reducing antioxidant power.

**Table 3:** Effects of Kratom leaves supplement on feed intake and performance of fattening goats.

Parameters	Supplement Levels of DKTL (g/d) ^1^				SEM ^2^	Contrasts *p*-Value ^3^	
	T1	T2	T3	T4		L	Q
Day on test	90	90	90	90	-		
Pen replicates	5	5	5	5	-		
Initial BW, kg	17.7	17.7	17.7	17.7	0.55	1.00	1.00
Final BW, kg	30.1	30.2	31.8	31.3	1.14	0.59	0.89
Weight gain (kg)	12.4	12.5	14.1	13.6	1.11	0.29	0.78
DMI							
kg/d	0.731	0.717	0.767	0.769	0.03	0.46	0.87
% BW	3.10	3.01	3.10	3.15	0.09	0.62	0.51
g/kg of BW^0.75^	68.1	66.4	69.03	69.77	1.96	0.36	0.51
**Nutrient Intake, kg/** **d ^4^**							
OMI, kg/d	0.693	0.68	0.727	0.729	0.02	0.46	0.87
CPI, kg/d	0.119	0.116	0.124	0.125	0.01	0.46	0.87
NDFI, kg/d	0.339	0.333	0.356	0.357	0.01	0.46	0.87
ADFI, kg/d	0.161	0.158	0.168	0.169	0.005	0.47	0.86
ADG, kg/d	0.138	0.139	0.157	0.151	0.14	0.90	0.88
ADG, g/kg BW^0.75^	13.03	12.92	14.19	13.65	0.95	0.48	0.83
G:F, kg/kg	0.191	0.194	0.206	0.196	0.01	0.67	0.63
FCR	5.51	5.25	4.88	5.14	0.42	0.44	0.56

^1^ T1 = 0.00, T2 = 2.22, T3 = 4.44, T4 = 6.66 g/goat per day of dried Kratom leaves (DKTL). ^2^ SEM = standard error of the mean. ^3^ Significant at *p* < 0.05 and were considered trends when *p* < 0.10. ^4^ OMI = organic matter intake, CPI = crude protein intake, NDFI = neutral detergent fiber intake, ADFI = acid detergent fiber intake, ADG = average daily gain, BW^0.75^ = metabolic weight, G:F = gain per feed ratio, FCR = feed conversion ratio.

**Table 4:** Effects of Kratom leaves supplement on slaughtered carcass characteristics of fattening goats.

Parameters	Supplement Levels of DKTL (g/d) ^1^				SEM ^2^	Contrasts *p*-Value ^3^	
	T1	T2	T3	T4		L	Q
Shrunk live weight, kg	33.83	32.67	34.17	35.5	1.19	0.38	0.45
HCW ^4^, kg	15.33	14.83	15.67	16.33	0.38	0.21	0.39
Warm dressing percentage, %	45.33	45.49	45.86	46.11	1.39	0.64	0.97
CCW ^5^, kg	14.83	14.7	15.4	16.1	0.41	0.14	0.52
Cold dressing percentage, %	43.85	45.05	45.08	45.45	1.07	0.30	0.68
Carcass length, cm	63.97	64.0	63.2	63.17	1.03	0.32	0.96
Carcass width, cm	14.6	16.33	16.83	16.53	0.35	0.01	0.02
LM area ^6^, cm^2^	11.43	11.23	15.1	14.77	0.72	<0.01	0.92
**Physical Properties of Meat Goats**							
WBSF ^7^ (kg/cm^2^)	2.92	3.10	3.41	3.59	0.15	<0.01	0.94
Drip loss (%)	2.68	2.87	1.57	1.72	0.29	0.03	0.95
Cooking loss (%)	33.98	37.36	25.31	26.44	3.07	0.02	0.69
**Ultimate pH ^8^**							
45 min pH	6.55	6.53	6.67	6.50	0.12	0.87	0.84
24 h pH	5.37	5.31	5.27	5.26	0.09	0.40	0.77
**Temperature ^9^**							
45 min	37.03	36.43	36.35	36.03	0.72	0.36	0.85
24 h	8.48	8.50	8.37	8.53	0.15	0.98	0.62
**Color of LM ^10^**							
L*	38.1	39.31	38.34	39.44	0.68	0.32	0.93
a*	16.16	18.42	17.06	17.77	0.63	0.20	0.20
b*	5.43	5.50	5.26	5.63	0.41	0.81	0.66

^1^ T1 = 0.00, T2 = 2.22, T3 = 4.44, T4 = 6.66 g/goat per day of dried Kratom leaves (DKTL). ^2^ SEM = standard error of the mean. ^3^ Significant at *p* < 0.05 and were considered trends when *p* < 0.10. ^4^ HCW = hot carcass weight. ^5^ CCW = cold carcass weight. ^6^ LM = Longissimus muscle area, cm^2^ from *Longissimus dorsi*. ^7^ WBSF: Warner-Bratzler shear force. ^8^ pH measurements were taken 45 min after slaughter; pH measurements were taken 24 h after slaughter. ^9^ Temperature measurements were taken 45 min after slaughter; temperature measurements were taken at 24 h after slaughter. ^10^ L* values are a measure of lightness (higher value indicates a lighter color); a* values are a measure of redness (higher value indicates a redder color); b* values are a measure of yellowness (higher value indicates a more yellow color), by CIE = Complete international commission on illumination (Hunter color flex).

**Table 5:** Effects of Kratom leaves supplement on carcass composition of fattening goats.

Parameters	Supplement Levels of DKTL (g/d) ^1^				SEM ^2^	Contrasts *p*-Value ^3^	
	T1	T2	T3	T4		L	Q
Carcass composition, kg							
Loins (kg)	0.503	0.567	0.650	0.647	0.07	0.11	0.62
Hind leg (kg)	1.723	1.780	1.747	1.857	0.09	0.33	0.74
Chump (kg)	0.990	0.923	0.827	1.000	0.05	0.74	0.02
Rack (kg)	0.677	0.800	0.667	0.647	0.19	0.77	0.68
Shoulder (kg)	1.470	1.487	1.517	1.650	0.1	0.31	0.63
Fore leg (kg)	1.510	1.600	1.617	1.597	0.07	0.4	0.45
Breast (kg)	0.423	0.490	0.487	0.493	0.04	0.39	0.57
Neck (kg)	0.717	0.700	0.500	0.707	0.09	0.56	0.22
**Carcass Composition ^4^, %**							
Loins, %	6.29	6.77	8.11	7.52	0.87	0.18	0.5
Hind leg, %	21.51	21.46	21.82	21.61	1.04	0.88	0.94
Chump, %	12.37	11.04	10.33	11.62	0.6	0.25	0.04
Rack, %	8.45	9.59	8.32	7.53	2.34	0.67	0.65
Shoulder, %	18.36	17.82	18.94	19.20	1.2	0.58	0.78
Fore leg, %	18.83	19.16	20.17	18.59	0.84	0.94	0.29
Breast, %	5.29	5.86	6.10	5.74	0.46	0.57	0.47
Neck, %	8.92	8.37	6.27	8.22	1.14	0.38	0.26

^1^ T1 = 0.00, T2 = 2.22, T3 = 4.44, T4 = 6.66 g/goat per day of dried Kratom leaves (DKTL). ^2^ SEM = standard error of the mean. ^3^ Significant at *p* < 0.05 and were considered trends when *p* < 0.10. ^4^ Carcass composition = as a percentage of chilled carcass weight.

**Table 6:** Effects of Kratom leaves supplement on chemical composition and physical properties of *Longissimus dorsi* muscle of fattening goats.

Parameters	Supplement Levels of DKTL (g/d) ^1^				SEM ^2^	Contrasts *p*-Value ^3^	
	T1	T2	T3	T4		L	Q
Nutritional composition							
Dry matter, %	23.32	24.10	25.12	24.02	0.51	0.18	0.08
Ash, %	1.55	1.50	1.65	1.63	0.11	0.55	0.57
Protein, %	20.77	21.67	22.86	23.07	0.32	<0.01	0.34
Ether extract, %	9.26	9.25	8.97	3.88	1.50	0.03	0.10
Calcium, %	0.95	0.11	0.10	0.11	0.01	0.81	0.81
Phosphorous, %	0.65	0.63	0.65	0.66	0.04	0.71	0.23

^1^ T1 = 0.00, T2 = 2.22, T3 = 4.44, T4 = 6.66 g/goat per day of dried Kratom leaves (DKTL). ^2^ SEM = standard error of the mean. ^3^ Significant at *p* < 0.05 and were considered trends when *p* < 0.10.

**Table 7:** Effects of Kratom leaves supplement on fatty acid (FA) profiles (% of total FA) in *Longissimus dorsi* muscle of fattening goats.

Parameters	Supplement Levels of DKTL (g/d) ^1^				SEM ^2^	Contrasts *p*-Value ^3^	
	T1	T2	T3	T4		L	Q
Fatty acid, % of total FAME ^4^							
C12:0	0.33	0.29	0.30	0.32	0.04	0.91	0.41
C14:0	2.59	2.74	2.45	2.78	0.11	0.63	0.49
C14:1	0.36	0.40	0.23	0.85	0.08	<0.01	<0.01
C16:0	25.11	22.52	23.13	23.43	0.32	<0.011	<0.01
C16:1	1.72	2.12	1.87	2.45	0.24	0.09	0.73
C18:0	15.42	13.72	14.17	14.42	0.26	0.05	<0.01
C18:1 n9	42.04	45.90	45.88	45.40	0.22	<0.01	<0.01
C18:2 n6	7.41	7.09	7.03	5.41	0.34	<0.01	0.08
C18:3 n3	3.02	3.23	3.15	3.01	0.15	0.89	0.27
n6/n3	2.45	2.22	2.23	1.80	0.16	0.02	0.44
C20:0	0.63	0.39	0.38	0.33	0.03	<0.01	0.01
C20:4 n6	1.13	0.98	1.00	1.10	0.12	0.87	0.32
C20:5 n3	0.25	0.64	0.43	0.49	0.05	0.06	0.01
SFA ^5^	44.08	39.65	40.42	41.29	0.33	<0.01	<0.01
MUFA ^6^	44.11	48.42	47.97	48.70	0.36	<0.01	<0.01
PUFA ^7^	11.81	11.94	11.61	10.01	0.35	<0.01	0.03
PUFA/SFA	0.27	0.30	0.29	0.24	0.01	0.07	<0.01

^1^ T1 = 0.00, T2 = 2.22, T3 = 4.44, T4 = 6.66 g/goat per day of dried Kratom leaves (DKTL). ^2^ SEM = standard error of the mean. ^3^ Significant at *p* < 0.05 and were considered trends when *p* < 0.10. ^4^ FAME = fatty acid methyl esters. Fatty acid profile: C12:0 = Lauric acid, C14:0 = Myristic acid, C14:1 = Myristoleic acid, C16:0 = Palmitic acid, C16:1 = Palmitoleic acid, C18:0 = Stearic acid, C18:1 n9 = Oleic acid, C18:2 n6 = Linoleic acid, C18:3 n3 = α-Linolenic acid, n6/n3 = omega 6 per omega 3 ratio, C20:0 = Arachidic acid, C20:4 n6 = Arachidonic acid, C20:5 n3 = Eicosapentaenoic acid, ^5^ SFA = short chain fatty acid. ^6^ MUFA = mono-unsaturated fatty acid. ^7^ PUFA = poly-unsaturated fatty acid.

**Table 8:** Effects of Kratom leaves supplement on hematology parameters of fattening goats.

Parameters	Supplement Levels of DKTL (g/d) ^1^				SEM ^2^	Contrasts *p*-Value ^3^	
	T1	T2	T3	T4		L	Q
TP ^4^, g%							
0 h, post-feeding	5.89	6.45	6.14	6.26	0.42	0.66	0.60
4 h, post-feeding	6.13	5.99	6.20	6.18	0.16	0.71	0.76
Mean	6.01	6.22	6.17	6.22	0.27	0.66	0.79
ALB ^5^, g%							
0 h, post-feeding	3.67	3.58	3.70	3.77	0.07	0.20	0.28
4 h, post-feeding	3.58	3.56	3.79	3.72	0.09	0.11	0.71
Mean	3.62	3.57	3.75	3.74	0.06	0.07	0.71
GLB ^6^, g%							
0 h, post-feeding	2.22	2.87	2.44	2.50	0.36	0.81	0.42
4 h, post-feeding	2.56	2.43	2.40	2.46	0.17	0.76	0.67
Mean	2.39	2.65	2.42	2.48	0.24	0.97	0.71
A:G ratio ^7^, g%							
0 h, post-feeding	1.66	1.36	1.53	1.52	0.15	0.69	0.33
4 h, post-feeding	1.45	1.48	1.58	1.53	0.11	0.62	0.78
Mean	1.56	1.42	1.55	1.53	0.11	0.92	0.69
SGOT ^8^, U/L							
0 h, post-feeding	102.33	86.00	104.67	105.67	6.73	0.47	0.33
4 h, post-feeding	113.67	91.33	113.33	109.33	4.71	0.76	0.18
Mean	108.00	88.67	109.00	107.50	5.20	0.57	0.24
SGPT ^9^, U/L							
0 h, post-feeding	26.00	25.33	20.67	22.00	2.41	0.29	0.77
4 h, post-feeding	26.67	21.67	21.67	23.33	1.30	0.33	0.16
Mean	26.33	23.50	21.17	22.67	1.55	0.27	0.41
ALP ^10^, U/L							
0 h, post-feeding	398.00	292.00	229.33	334.33	0.48	0.14	0.68
4 h, post-feeding	442.67	349.33	257.00	346.33	0.18	0.08	0.40
Mean	420.33	320.67	243.17	340.33	0.33	0.11	0.55

^1^ T1 = 0.00, T2 = 2.22, T3 = 4.44, T4 = 6.66 g/goat per day of dried Kratom leaves (DKTL). ^2^ SEM = standard error of the mean. ^3^ Significant at *p* < 0.05 and were considered trends when *p* < 0.10. ^4^ TP = total protein, ^5^ ALB = albumin, ^6^ GLB = globulin, ^7^ A/G ratio = albumin to globulin ratio, ^8^ SGOT = Serum glutamic oxaloacetic transaminase, ^9^ SGPT = Serum glutamate-pyruvate transaminase, ^10^ ALP = alkaline phosphatase.

**Table 9:** Effects of Kratom leaves supplement on hematology parameters of fattening goats (continue).

Parameters	Supplement Levels of DKTL (g/d) ^1^				SEM ^2^	Contrasts *p*-Value ^3^	
	T1	T2	T3	T4		L	Q
RBC ^4^, 10^6^/µL							
0 h, post-feeding	3.52	3.10	3.52	3.56	0.23	0.63	0.36
4 h, post-feeding	3.48	3.40	3.62	3.54	0.25	0.73	1.00
Mean	3.50	3.25	3.57	3.55	0.24	0.68	0.66
Hb ^5^, g/dL							
0 h, post-feeding	10.73	10.57	10.53	10.37	0.48	0.58	1.00
4 h, post-feeding	11.37	11.47	10.73	10.40	0.24	<0.01	0.36
Mean	11.05	11.02	10.63	10.38	0.31	0.09	0.70
MCV ^6^, fL							
0 h, post-feeding	86.33	94.43	80.77	80.57	0.12	0.33	0.09
4 h, post-feeding	91.57	93.83	78.67	81.03	0.10	0.99	0.14
Mean	88.95	94.13	79.72	80.80	0.09	0.63	0.09
MCH ^7^, pg							
0 h, post-feeding	30.63	34.43	30.10	29.17	0.22	0.19	0.16
4 h, post-feeding	33.30	34.07	29.83	29.37	0.21	0.76	0.34
Mean	31.97	34.25	29.97	29.27	0.22	0.42	0.23
MCHC ^8^, g/dL							
0 h, post-feeding	35.40	36.47	37.20	36.20	0.56	0.22	0.70
4 h, post-feeding	36.30	36.33	37.87	36.30	0.41	0.31	0.20
Mean	35.85	36.40	37.53	36.25	0.39	0.19	0.32
RDW-CV ^9^, %							
0 h, post-feeding	28.03	27.10	27.87	27.83	0.67	0.46	0.37
4 h, post-feeding	27.93	28.33	28.03	27.90	0.95	0.64	0.73
Mean	27.98	27.72	27.95	27.87	0.99	0.87	0.74
WBC ^10^, 10^3^/µL							
0 h, post-feeding	15.96	17.74	12.79	14.99	0.72	0.03	0.77
4 h, post-feeding	15.30	17.57	13.94	15.90	1.14	0.70	0.88
Mean	15.63	17.65	13.37	15.45	0.80	0.17	0.97
NEU ^11^, %							
0 h, post-feeding	33.33	28.67	29.33	36.67	0.92	0.49	0.97
4 h, post-feeding	62.00	48.67	50.00	46.67	0.25	0.46	0.52
Mean	47.67	38.67	39.67	41.67	0.78	0.43	0.77
LYMPH ^12^, %							
0 h, post-feeding	64.00	57.00	60.33	54.67	0.86	0.93	0.58
4 h, post-feeding	34.00	44.33	41.00	42.67	0.85	0.66	0.67
Mean	49.00	50.67	50.67	48.67	1.00	0.82	0.99
MONO ^13^, %							
0 h, post-feeding	1.67	1.67	1.33	1.00	0.67	0.69	0.86
4 h, post-feeding	3.67	4.00	3.00	4.00	0.98	0.87	0.71
Mean	2.67	2.83	2.17	2.50	0.98	0.94	0.70

^1^ T1 = 0.00, T2 = 2.22, T3 = 4.44, T4 = 6.66 g/goat per day of dried Kratom leaves (DKTL). ^2^ SEM = standard error of the mean. ^3^ Significant at *p* < 0.05 and were considered trends when *p* < 0.10. ^4^ RBC = red blood cell; ^5^ Hb = hemoglobin; ^6^ MCV = mean corpuscular volume; ^7^ MCH = mean corpuscular hemoglobin; ^8^ RDW = RBC distribution width; ^9^ MCHC = mean corpuscular hemoglobin concentration; ^10^ WBC, white blood cells; ^11^ NEU, neutrophil; ^12^ LYMPH, lymphocytes; ^13^ MONO, monocytes.

**Table 10:** Effects of Kratom leaves supplement on plasma parameters of fattening goats.

Parameters	Supplement Levels of DKTL (g/d) ^1^				SEM ^2^	Contrasts *p*-Value ^3^	
	T1	T2	T3	T4		L	Q
CHOL, mg%							
0 h, post-feeding	80.00	77.33	70.00	75.33	8.14	0.59	0.55
4 h, post-feeding	84.00	78.33	67.33	71.33	10.30	0.26	0.60
Mean	82.00	77.83	68.67	73.33	9.77	0.40	0.63
HDL-Chol, mg%							
0 h, post-feeding	38.67	38.67	38.00	39.00	4.41	0.99	0.90
4 h, post-feeding	40.33	48.00	44.33	43.33	6.03	0.83	0.44
Mean	39.50	43.33	41.17	41.17	5.05	0.89	0.68
TG, mg%							
0 h, post-feeding	39.00	39.00	29.00	45.67	7.63	0.82	0.40
4 h, post-feeding	60.33	57.33	37.67	49.33	8.54	0.35	0.56
Mean	49.67	48.17	33.33	47.50	7.86	0.66	0.47
LDL-Chol, mg%							
0 h, post-feeding	33.87	26.77	27.93	29.20	5.28	0.55	0.39
4 h, post-feeding	39.40	35.53	30.40	31.47	4.77	0.17	0.58
Mean	36.63	31.15	29.17	30.33	4.60	0.28	0.43

^1^ T1 = 0.00, T2 = 2.22, T3 = 4.44, T4 = 6.66 g/goat per day of dried Kratom leaves (DKTL). ^2^ SEM = standard error of the mean. ^3^ Significant at *p* < 0.05 and were considered trends when *p* < 0.10. CHOL = cholesterol; HDL = high-density lipoprotein; LDL = Low-density lipoprotein; TG = triglyceride.

## Data Availability

Not applicable.
